# Abundance of conserved CRISPR-Cas9 target sites within the highly polymorphic genomes of *Anopheles* and *Aedes* mosquitoes

**DOI:** 10.1038/s41467-020-15204-0

**Published:** 2020-03-18

**Authors:** Hanno Schmidt, Travis C. Collier, Mark J. Hanemaaijer, Parker D. Houston, Yoosook Lee, Gregory C. Lanzaro

**Affiliations:** 1 Vector Genetics Laboratory, Department of Pathology, Microbiology, and Immunology, School of Veterinary Medicine, University of California, Davis, CA 95616 USA; 2 Present Address: Winclove Probiotics, Hulstweg 11, 1032 LB Amesterdam, Netherlands

**Keywords:** Synthetic biology, Functional genomics, CRISPR-Cas9 genome editing

## Abstract

A number of recent papers report that standing genetic variation in natural populations includes ubiquitous polymorphisms within target sites for Cas9-based gene drive (CGD) and that these “drive resistant alleles” (DRA) preclude the successful application of CGD for managing these populations. Here we report the results of a survey of 1280 genomes of the mosquitoes *Anopheles gambiae*, *An. coluzzii*, and *Aedes aegypti* in which we determine that ~90% of all protein-encoding CGD target genes in natural populations include at least one target site with no DRAs at a frequency of ≥1.0%. We conclude that the abundance of conserved target sites in mosquito genomes and the inherent flexibility in CGD design obviates the concern that DRAs present in the standing genetic variation of mosquito populations will be detrimental to the deployment of this technology for population modification strategies.

## Abstract (web summary)

Genetic variation in natural populations could represent gene drive resistant alleles, preventing successful application for population management. Here the authors survey 1280 genomes from three mosquito species and concludes natural variation will not be detrimental to deploying gene drive technology.

## Introduction

The discovery of clustered regularly interspaced short palindromic repeats (CRISPR) in bacteria^1,2^ and the Cas9 enzyme (CRISPR associated protein 9)^3–5^, has revolutionized our capacity to genetically engineer a wide range of organisms. The subsequent development of CRISPR-Cas9-based gene drives^6^ has further increased the potential application of this technology. Gene drives promote the spread of introduced genetic elements (e.g., alternative alleles, exogenous genes) through populations by altering the way in which they are inherited, such that the desired genetic element is over-represented among progeny (“Super-Mendelian inheritance”)^7^. This leads to an increase in frequency of the introduced genetic element, potentially until fixation in the targeted population.

One application of CRISPR-Cas9 gene drive that has gained a great deal of attention is the possibility of controlling populations of disease vectors like mosquitoes. The focus of current efforts is *Anopheles gambiae* and *An. coluzzii* which transmit malaria, and *Aedes aegypti* which transmits dengue, chikungunya, yellow fever, and Zika. Collectively, these diseases cause hundreds of thousands of human deaths per year^8^. New strategies for controlling these vectors are sorely needed because currently available control methods are costly, increasingly ineffective due to insecticide resistance^9^ and are generally difficult to deploy in rural endemic areas. Alternative genetic-based strategies for vector control are not new, however, the recent advances in genetic engineering and gene drive have sparked increased interest in this approach. There are two broad categories of strategies involving genetically engineered mosquitoes (GEM) with gene drive currently under development: population suppression aimed at greatly reducing or eliminating the mosquito population^10^ and population modification, which renders mosquitoes incapable of transmitting a pathogen but otherwise leaves it unaltered^11^. Recently, CRISPR-Cas9-based gene drive systems have been designed for population modification in *Anopheles*^12^ and *Aedes*^13^ and for population suppression in *Anopheles*^10,14^ mosquitoes.

Experiments demonstrating the capacity of gene-drive constructs to spread through wild-type populations in laboratory cages have yielded promising results^10^. A major limitation of these experiments is that they use populations of mosquitoes derived from long-standing laboratory colonies that do not replicate populations as they occur in nature^15,16^. Specifically, founder effects during establishment, repeated bottlenecks experienced during maintenance, and selection for adaptation to the laboratory environment in these colonies all result in the loss of genetic variability relative to their counterparts in nature^17–19^.

Recently, several population genomic studies have amassed a large volume of genomic data from natural populations of *An. gambiae*^20,21^*, An. coluzzii*^21^, and *Ae. aegypti*^22^. These surveys revealed exceptionally high levels of genetic variability leading some authors to warn that CRISPR-Cas9-based gene-drive systems (CGD) may be prone to failure due to drive resistance resulting from standing genetic variation. This includes uncleavable alleles within the target sequence that are not recognized by the guide RNA^21,23^. A study of the impact of drive resistance alleles (DRAs) on the performance of CGD in natural populations of the flour beetle, *Tribolium castaneum*, concluded that population-specific rare alleles will probably reduce or eliminate drive efficacy^24^. General modeling approaches revealed that standing genetic variation could even exceed de novo mutations in contributing to CGD resistance^25^. Given the interest in the development of CGD, a systematic evaluation of the distribution of polymorphisms within the genomes of these critical mosquito species and its impact on potential target sites for CRISPR-Cas9 editing is warranted.

Here we present genome-wide screens of the three principal human disease vector species *An. gambiae*, *An. coluzzii*, and *Ae. aegypti* for the presence of CRISPR-Cas9 target sites and an analysis of the degree of polymorphism therein. In detail, we search all transcribed regions of protein-coding genes in the species’ reference genomes for potential CRISPR-Cas9 target sites. We then subject each target site to a screen for nucleotide polymorphisms (single nucleotide polymorphisms, insertions, deletions) in the genomes of mosquitoes sampled directly from natural populations. Our analyses include 111 *An. gambiae*, 100 *An. coluzzii*, and 132 *Ae. aegypti* genomes from our lab plus publicly available polymorphism data from 937 additional *An. gambiae s.l*. samples. The special interest in *An. gambiae* as the principal vector of malaria in Africa results in a larger number of individual mosquito sequence data compared with any other mosquito species. Additional insights gained from including the larger number of sequences compared with *An. coluzzii* and *Ae. aegypti* outweigh the benefits of having equal numbers per species. We find that >30% of protein-coding genes have potential CRISPR-Cas9 targets with GC content between 30 and 70% and no off-target sequence. This drops to 8.4% if sites with DRAs at frequencies >1% in natural populations are excluded. Nonetheless ~90% of all protein-coding genes contain at least one target site that remain after this filtering. Based on these observations we conclude that DRAs within the standing variation that exists in natural populations of the mosquito species studied will not pose a problem to the successful deployment of CRISPR-Cas9-based gene drive for population modification strategies. Gene drive used as part of population suppression strategies are more likely to be unsustainable because of the presence of low-frequency DRAs and the fact that they impose much stronger selection favoring them.

## Results

### Identifying potential CRISPR-Cas9 target sites

We began our analysis by identifying all potential CRISPR-Cas9 guide RNA (gRNA) target sites in each species’ genomes and subjecting each to an analysis to identify DRAs. We define potential target sites as 23 bp stretches with the nucleotides ‘NGG’ at one 3′-end (NGG = protospacer adjacent motif, PAM), located in a transcript of a protein-coding gene. To make the analysis more conservative, we restricted our search to target sites with a GC content between 30 and 70% and no close (<4 mismatches) sequence matching anywhere else in the genome that could produce off-target activity. The total number of potential target sites was estimated by screening the latest versions of the publicly available reference genomes of *An. gambiae* (AgamP4) and *Ae. aegypti* (AaegL4) using the program CHOPCHOP^26–28^. The AgamP4 genome is sequenced from an *An. gambiae*–*An. coluzzii*-hybrid laboratory strain and is suitable as a reference for both, *An. coluzzii* and *An. gambiae*^29^.

**Fig. 1:**
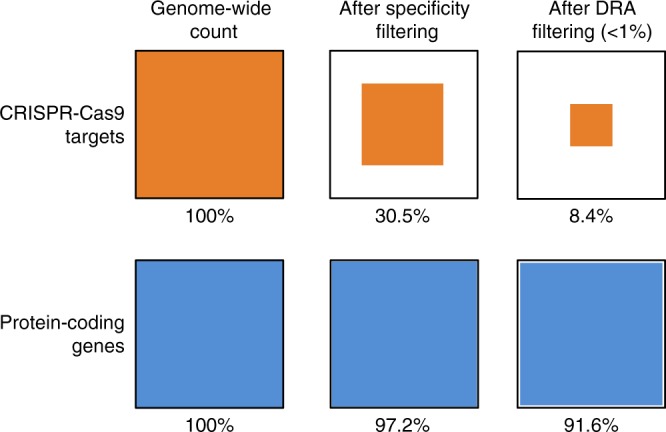
Sketch of the effect of quality filtering on the number of “good” targets/genes. Genome-wide count of CRISPR-Cas9 targets (orange) and protein-coding genes (blue) is set to 100% each. During specificity filtering (GC content between 30 and 70% and no off-targets) and DRA filtering (DRA frequency <1%), the number of available targets drops well below 10% (Table 2). Nevertheless, ~90% of all protein-coding genes still contain at least one good target. Colored areas correspond to the values for the combined data of *An. gambiae* and *An. coluzzii*. The percentages are similar for *Ae. aegypti* (not shown). Source data are provided as a Source Data file.

We identified 1,196,509 high-quality potential targets in the genome of *An. gambiae s.l*. and 828,454 for *Ae. aegypti* (Table 1). While 69.5% (*An. gambiae s.l*.) and 77.2% (*Ae. aegypti*) of the raw target sites were dismissed during quality filtering, the overwhelming majority of coding genes contain at least one potential CRISPR-Cas9 target site (97.2% in *An. gambiae s.l*. and 92.2% in *Ae. aegypti*, Fig. 1).

**Table 1:** Target sites for CRISPR-Cas9 editing in mosquito genomes.

	*Anopheles gambiae s.l*. (AgamP4.11)	*Aedes aegypti* (AaegL5.1)
Genome size (Mbp)	230.5	1195
Coding part of the genome (Mbp)	25.7 (11.2%)	59.1 (5%)
Raw targets	3,918,579	3,638,628
Potential targets	1,196,509 (30.5%)	828,454 (22.8%)
Protein-coding genes	12,562	13,601
Protein-coding genes with potential targets	12,213 (97.2%)	12,536 (92.2%)

Raw targets are all unique target sites suitable for CRISPR-Cas9 editing found in transcripts of protein-coding genes. Potential targets refer to sites that passed filtering for off-target effects and GC content. The same reference genome (AgamP4.11) was used for *A. gambiae* and *A. coluzzii*.

*Mbp* mega base pairs.

### DRA frequencies in natural mosquito populations

We conducted screening of potential target sites for DRAs using five unfiltered datasets of nucleotide polymorphisms from natural mosquito populations (Table 2) from our lab (Vector Genetics Laboratory; VGL) and The *Anopheles gambiae* 1000 Genomes Consortium (Ag1000G). The nucleotide diversity (*π)* of transcripts in protein-coding genes was between 0.94 and 1.02% (Table 2, Supplementary Table 1). For comparison, a relatively high estimate of human nucleotide diversity is 0.6%^30^.

**Table 2:** Effect of polymorphisms on potential targets.

	*An. gambiae* VGL	*An. gambiae* Ag1000G	*An. coluzzii* VGL	*An. coluzzii* Ag1000G	*Ae. aegypti* VGL
Samples	111	654	100	283	132
Nucleotide diversity (π) in transcribed regions of protein-coding genes	0.98%	1.02%	1%	0.95%	0.94%
Good targets (% of raw targets/% of potential targets)	216,793 (5.5%/18.1%)	34,995 (0.9%/2.9%)	174,117 (4.4%/14.6%)	93,142 (2.4%/7.8%)	273,627 (7.5%/33%)
Protein-coding genes with good targets, i.e., no variation at all (% of all protein-coding genes)	11,163 (88.9%)	4281 (34.1%)	10,832 (86.2%)	8796 (70%)	12,536 (87.5%)
Protein-coding genes with good targets, i.e., no variation at frequencies ≥1% (% of all protein-coding genes)	11,721 (93.3%)	11,525 (91.3%)	11,415 (90.9%)	11,540 (91.9%)	12,096 (88.9%)

The variant data comprises unfiltered calls for the three mosquito species. Sequence data was taken from the UC Davis Vector Genetics Lab archive. We also included publicly available variant data for *An. gambiae* and *An. coluzzii* from the Ag1000G project. Good target sites are potential target sites that have been additionally filtered for variant data, i.e., target sites that are suitable for gene editing with a high probability of showing no resistance alleles in natural populations.

### DRA frequencies and abundance of good CRISPR-Cas9 targets

**Fig. 2:**
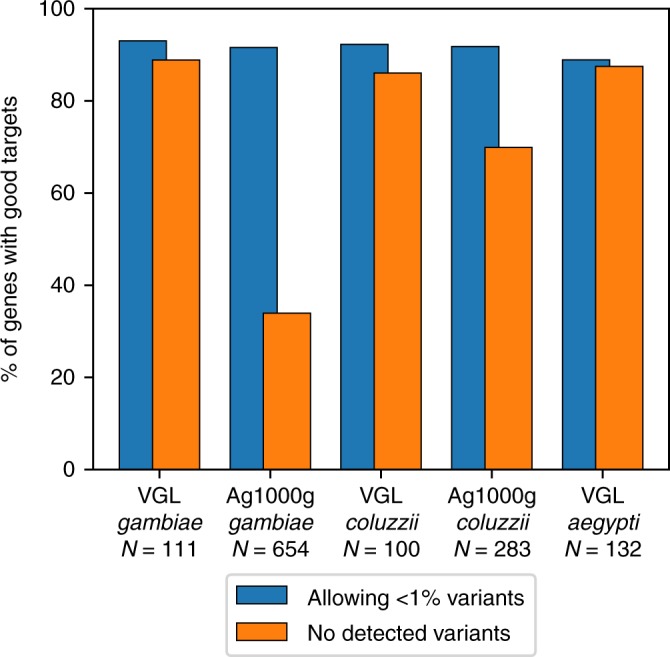
Frequency of genes with good targets. The fraction of genes with good targets is dependent on the presence of low-frequency DRAs. When dismissing all targets with DRA frequencies > 0.0, the fraction of genes with good targets decreases with increasing sample size. Ignoring DRAs with frequencies below 1% in the dataset results in ~90% of genes having at least one good target in all datasets examined. VGL: Vector Genetics Laboratory, Ag1000G: The *Anopheles gambiae* 1000 Genomes Consortium. Source data are provided as a Source Data file.

We define a “good” CRISPR-Cas9 target site as a potential target which contains no DRAs above a predefined DRA threshold frequency. Sample size limits our ability to detect DRAs below a certain frequency. If we set the threshold frequency at 0.00 the proportion of good targets is highly dependent on sample size, with an ordinary least-squares coefficient of determination (*r*^2^) of 0.99 (Fig. 2, orange bars). However, setting the DRA threshold at 0.01 essentially eliminates this sampling effect (*r*^2^ of 0.00) If this threshold (<1%) is applied to all five datasets we find ~90% of protein-coding genes contain at least one good target (Fig. 2, blue bars).

**Fig. 3:**
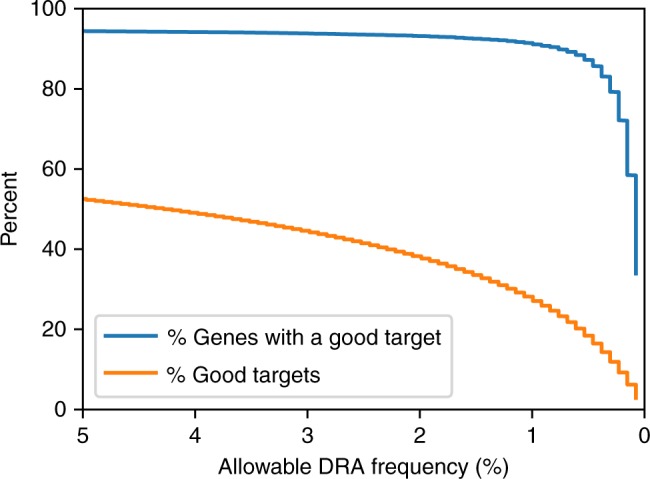
Effects of polymorphisms on targets. Percent of genes containing at least one good target (blue) and percent of good targets out of possible targets as a function of DRA frequency threshold (orange). This analysis is based on *N* = 654 *An. gambiae* samples (Ag1000G data). DRA frequency threshold is the value beyond which alternative alleles are considered to be DRAs and are filtered out (i.e., a DRA frequency threshold of 0.01 means, alternative alleles with a frequency below 1% are ignored during filtering). Note the constant decline in the fraction of good targets (orange), which is not mirrored in the fraction of genes containing good targets (blue) until the DRA frequency threshold is set at <0.01. Source data are provided as a Source Data file.

The relationship between the DRA threshold frequency and the percentage of genes containing at least one good target is illustrated in detail for the Ag1000G *An. gambiae* dataset (*N* = 654) (Fig. 3). The chance that any specific target site will be free of DRAs is much lower than the chance that a given gene will contain at least one good target. Only 28.2% of specific targets are free from DRAs with ≥1% frequency, dropping to 6.3% for variants with a frequency of 0.15%. Less than 3% of potential targets are completely free of observed DRAs.

The fraction of protein-coding genes containing at least one good target is largely independent of the DRA frequency threshold down to a threshold of ~1%, at which point it drops steeply (Fig. 3, blue line). For example, 91.6% of protein-coding genes contain a good target, with no DRAs at frequencies ≥1%. However, only 58.5% of protein-coding genes contain at least one good target when the DRA threshold is set at 0.15%. The fraction of good targets among all potential targets is far more sensitive to the DRA frequency threshold, declining steadily as the DRA frequency threshold is decreased (Fig. 3, orange line).

## Discussion

In this study we confirm what has been widely reported that mosquitoes in the genera *Aedes* and *Anopheles* have genomes that are highly polymorphic^21,22^. The suggestion that standing genetic variation will render Cas9-based gene drives ineffective in natural mosquito populations seems to be plausible. Indeed, <3% of potential high-quality Cas9 targets are free from any observed variation and increasing the sample size analyzed will only reduce this value. However, protein-coding genes almost always contain many potential targets. The median number of potential targets per coding gene for *An. gambiae* and *An. coluzzii* is 72 and for *Ae. aegypti* it is 47. Even if there is only a 3% chance that any individual target will be a good target, with 72 options the chance that at least one will be good is 89% (Fig. 1). Consequently, our analyses show that ~90% of all protein-coding genes have conserved target sites for CRISPR-Cas9 editing in all three species examined. The broad similarity of results between the two *Anopheles* species and *Ae. aegypti*, which has quite different genomic characteristics, suggests the observed pattern represents a general principle. This could be tested in the future by examining population genomic data from additional species.

These results have implications for evaluating the prospects of population modification versus population suppression strategies. GEMs for population suppression are designed to eliminate fertile females from a target population and so transgenic individuals obviously have extremely low fitness^31^. Very strong selection favoring a wild-type genotype may be to some extent countered by the self-replicating gene drive. However, every individual with a genotype that includes a DRA will be subjected to strong positive selection. Therefore, even low-frequency DRAs (including private nucleotide polymorphisms) pose a high risk to population suppression strategies, since DRAs would rapidly increase in frequency^32,33^. This translates to establishing a DRA threshold near zero. As we demonstrate here, this will reduce the number of protein-coding genes that are useful candidates for genetic engineering (Fig. 3), especially in large natural populations as depicted by the sample size dependence in fraction of genes with targets having no polymorphisms at all (Fig. 2). A scenario where this might be useful is the targeting of small, defined populations, where spillover to neighboring populations is unwanted and can be avoided by the intended use of alleles that are fixed in the target population but absent in the neighbor population^34,35^. While this can also be applied with population modification strategies, these also can be specifically designed to have a negligible fitness cost relative to wild-type^12^. In this case, low frequency wild-type genotypes that include a DRA should not affect the gene drive behavior detrimentally, since they would likely remain at low frequencies or be eliminated by drift^36^. Even highly efficient gene-drive systems generate DRAs at frequencies of ~1%, thus choosing this value as a threshold for standing variation is justified from the point of view that such a level of DRAs would have only marginal effects of inherent gene-drive performance.

When seeking to design a GEM with CGD for release into a natural population, researchers will most likely consider target sites excluded by the stringent filtering applied in this study. For example, we did not apply quality filtering of polymorphic sites in order to be as conservative as possible. In the planning of a GEM design study, researchers would clearly make an effort to exclude false positive calls, thereby reducing the total number/density of DRAs. Also, a single polymorphic site distant from the PAM (e.g., >−10 bp from PAM) is not expected to have dramatic effects on cleavage efficiencies. Moreover, different nucleotide positions in CRISPR-Cas9 target sites have quite unequal effects on cleavability^24,37^. In summary, when evaluating a specific candidate gene for GEM design, a much larger number of target sites for CRISPR-Cas9 editing could be considered for empirical evaluation.

The extensive amount of data analyzed for three of the most important human disease vector species in large parts of their distributional area present an unprecedented view of the feasibility of CRISPR-Cas9-based gene drives in mosquitoes. The results demonstrate that good target sites lacking DRAs or with DRAs present at low frequency are abundant in the three species studied. The abundance of good target sites in mosquito genomes and the inherent flexibility in CRISPR-Cas9-based gene-drive design suggests that drive resistance arising from selection on standing genetic variation will not be a detriment to the deployment of this technology for eliminating mosquito-borne diseases.

## Methods

### Search for potential CRISPR-Cas9 target sites

Potential CRISPR-Cas9 target sites were searched with the command line version of CHOPCHOP v6054ae8b29b9^26–28^ with Python v.2.7.15^38^, applying default settings and ‘Xu_2015’ efficiency scoring^39^. We modified CHOPCHOP slightly to fix a minor bug in the handling of chromosome names and to increase the maximum target (transcript) size. We restricted the search to the transcripts (coding sequences + untranslated regions) of protein-coding genes from the most recent annotation files downloaded from vectorbase.org, with 12,562 entries for protein-coding genes in *An. gambiae* (AgamP4.11) and 13,601 for *Ae. aegypti* (AaegL5.1). The output was filtered for targets that show no off-target sites with less than four mismatches to the original sequence and that have a GC content between 30 and 70%. We denote CRISPR-Cas9 target sites that passed this procedure as “potential target sites”.

### *Anopheles gambiae s.l.* data preparation

We used individual whole genome sequencing data from *N* = 111 *An. gambiae s.s*. samples from natural populations in Mali (*N* = 40), Cameroon (*N* = 5), Tanzania (*N* = 6), Zambia (*N* = 6), and the Comoro Islands (*N* = 54) and *N* = 100 *An. coluzzii* samples from natural populations in Mali (*N* = 66), Benin (*N* = 11), Equatorial Guinea (*N* = 3), Cameroon (*N* = 1), and São Tomé and Príncipe (*N* = 19). Specimens for sequencing were taken from the Vector Genetics Laboratory’s archive (Supplementary Data 1). Genomic DNA was sequenced on an Illumina HiSeq 4000 to a mean depth of 10.2X (Supplementary Table 2). Sequences were filtered for adapters with Trimmomatic v0.36^40^ and then mapped against the most current reference genome assembly ‘AgamP4’^41^ using BWA MEM v0.7.17-r1188^42^. Polymorphic sites were called with Freebayes v1.2.0^43^ applying default parameters but ‘theta=0.01’ and ‘max-complex-gap=3′.

The Ag1000G datasets were processed by MalariaGen (www.malariagen.net) using BWA-MEM mapping and GATK UnifiedGenotyper^44^ workflow^45^.

### *Aedes aegypti* data preparation

We used individual whole genome data from *N* = 132 *Ae. aegypti* samples from California (*N* = 122), Florida (*N* = 4), Mexico (*N* = 3), and South Africa (*N* = 3) from the Vector Genetics Laboratory’s archive (Supplementary Data 1) sequenced to a mean depth of 10.5X (Supplementary Table 2). Sequences were generated and data were processed in the same way as described above for *Anopheles* samples, using the most current reference genome assembly ‘AaegL5’^46^.

### Polymorphisms in potential CRISPR-Cas9 target sites

We did not include any subsequent quality filtering of detected polymorphisms to ensure having the broadest possible set of potential polymorphic sites and hence being as conservative as possible (i.e., removing all doubtful/undecidable potential CRISPR-Cas9 target sites). The potential CRISPR-Cas9 target sites were then filtered for polymorphic sites using custom scripts available at https://github.com/travc/Cas9_target_site_survey.

### Reporting summary

Further information on research design is available in the Nature Research Reporting Summary linked to this article.

## Supplementary information

Supplementary Information

Supplementary Data 1

Reporting Summary

Description of Additional Supplementary Files

## Data Availability

Sequence data sources are detailed in Supplemental Table S1. Data processing scripts and small datafiles are available in GitHub with the identifier: [10.5281/zenodo.3661448]^47^. Any additional data that support the findings of this study are available from the corresponding author upon reasonable request.
