# Supplementary material for: Abundance of conserved CRISPR-Cas9 target sites within the highly polymorphic genomes of Anopheles and Aedes mosquitoes
Source: Nat Commun. 2020 Mar 18;11:1425. doi: 10.1038/s41467-020-15204-0 (PMC7080748; doi:10.1038/s41467-020-15204-0)
Supplement: Supplementary file 1 — Supplementary Information [file 41467_2020_15204_MOESM1_ESM.docx]

## Supplementary Material to: “Abundance of conserved CRISPR-Cas9 target sites within highly polymorphic genomes of *Anopheles* and *Aedes* mosquitoes from natural populations”

## Hanno Schmidt, Travis C. Collier, Mark J. Hanemaaijer, Parker D. Houston, Yoosook Lee, Gregory C. Lanzaro

## **Supplementary Data 1 - Sample Information**

Information for all samples from the UC Davis Vector Genetics Laboratory’s (VGL) archive can be found in Supplementary Data S1 provided as a spreadsheet file.

Information for samples from The *Anopheles gambiae* 1000 Genomes Consortium (Ag1000G):

ftp://[ngs.sanger.ac.uk/production/ag1000g/phase2/AR1/samples/samples.meta.txt](http://ngs.sanger.ac.uk/production/ag1000g/phase2/AR1/samples/samples.meta.txt)

See the metadata file for additional info including country, site, collection year, and contributor.

See also: https://www.malariagen.net/data/ag1000g-phase-2-ar1

The 654 *An. gambiae* samples are labelled "S" and the 283 *An. coluzzii* samples "M" in the "m_s" column of the associated metadata.

## **Supplementary Table 1 - Nucleotide Diversity**

Shown is the nucleotide diversity (π) for each chromosome per dataset individually and for each full dataset. Upper part of the table provides values for protein-coding transcripts only (as used in the analysis) the lower part provides values for the whole genome for comparison. VGL-gam= *An. gambiae* data from the VGL archive (N=111), Ag1000G-gam= *An. gambiae* data from Ag1000G project (N=654), VGL-col= *An. coluzzii* data from the VGL archive (N=100), Ag1000G-col= An. coluzzii data from Ag1000G project (N=283), VGL-Aaeg= Ae. aegypti data from the VGL archive (N=132).

| Coding transcripts only | | | | | | |
| --- | --- | --- | --- | --- | --- | --- |
| chromosome | 2R | 2L | 3R | 3L | X | overall |
| VGL-gam | 0.009351 | 0.01176 | 0.011051 | 0.009892 | 0.003744 | 0.00984 |
| Ag1000G-gam | 0.009711 | 0.011909 | 0.011326 | 0.010464 | 0.00425 | 0.010184 |
| VGL-col | 0.009123 | 0.011878 | 0.011314 | 0.010246 | 0.004529 | 0.009985 |
| Ag1000G-col | 0.008473 | 0.011473 | 0.0109 | 0.01003 | 0.004019 | 0.009527 |
| chromosome | 1 | 2 | 3 |  |  | overall |
| VGL-Aaeg | 0.010524 | 0.009222 | 0.00879 |  |  | 0.009381 |
| Genome-wide (assuming all loci are accessible) | | | | | | |
| chromosome | 2R | 2L | 3R | 3L | X | overall |
| VGL-gam | 0.019412 | 0.024127 | 0.022617 | 0.019781 | 0.010248 | 0.020386 |
| Ag1000G-gam | 0.019025 | 0.022709 | 0.022449 | 0.020006 | 0.012148 | 0.020125 |
| VGL-col | 0.018923 | 0.024769 | 0.02343 | 0.020964 | 0.012279 | 0.020942 |
| Ag1000G-col | 0.016125 | 0.021551 | 0.020906 | 0.018967 | 0.010508 | 0.018338 |
| chromosome | 1 | 2 | 3 |  |  | overall |
| VGL-Aaeg | 0.012612 | 0.013883 | 0.013005 |  |  | 0.01326 |

## **Supplemental Table 2 - Sequencing Depth**

Values are calculated based on coding transcripts only. Dataset codes as in Supplementary Data S1. Std= standard deviation.

| chromosome | 2R | 2L | 3R | 3L | X | overall |
| --- | --- | --- | --- | --- | --- | --- |
| VGL-gam median | 8 | 8 | 8 | 8 | 8 | 8 |
| VGL-gam mean | 9.2 | 9 | 9.1 | 9 | 9.1 | 9.1 |
| VGL-gam std | 7 | 7 | 7 | 7 | 7.3 |  |
| chromosome | 2R | 2L | 3R | 3L | X | overall |
| Ag1000g-gam median | 31 | 30 | 31 | 30 | 28 | 30 |
| Ag1000g-gam mean | 32.1 | 32 | 32.4 | 31.6 | 29.7 | 31.8 |
| Ag1000g-gam std | 14.3 | 14.3 | 14.7 | 14.3 | 15 |  |
| chromosome | 2R | 2L | 3R | 3L | X | overall |
| VGL-col median | 11 | 10 | 10 | 10 | 10 | 10 |
| VGL-col mean | 11.6 | 11.3 | 11.4 | 11.2 | 11 | 11.4 |
| VGL-col std | 10.4 | 10.4 | 13.1 | 10.2 | 11.8 |  |
| chromosome | 2R | 2L | 3R | 3L | X | overall |
| Ag1000g-col median | 30 | 30 | 30 | 30 | 29 | 30 |
| Ag1000g-col mean | 30.3 | 30.1 | 30.4 | 29.7 | 29.3 | 30.1 |
| Ag1000g-col std | 10.6 | 10.5 | 10.5 | 10.4 | 11.2 |  |
| chromosome | 1 | 2 | 3 |  |  | overall |
| VGL-Aaeg median | 10 | 10 | 10 |  |  | 10 |
| VGL-Aaeg mean | 10.3 | 10.5 | 10.7 |  |  | 10.5 |
| VGL-Aaeg std | 7.1 | 7 | 7.1 |  |  |  |
