# Supplementary material for: Abundance of conserved CRISPR-Cas9 target sites within the highly polymorphic genomes of Anopheles and Aedes mosquitoes
Source: Nat Commun. 2020 Mar 18;11:1425. doi: 10.1038/s41467-020-15204-0 (PMC7080748; doi:10.1038/s41467-020-15204-0)
Supplement: Supplementary file 4 — Description of Additional Supplementary Files [file 41467_2020_15204_MOESM4_ESM.pdf]

**Title:** Supplementary Data 1

**Description:** Sample Information Information for all samples from the UC Davis Vector Genetics Laboratory's (VGL) archive can be found in Supplementary Data 1 provided as a spreadsheet file. Information for samples from The Anopheles gambiae 1000 Genomes Consortium (Ag1000G): ftp://ngs.sanger.ac.uk/production/ag1000g/phase2/AR1/samples/samples.meta.txt See the metadata file for additional info including country, site, collection year, and contributor. See also: https://www.malariagen.net/data/ag1000g-phase-2-ar1 The 654 An. gambiae samples are labelled "S" and the 283 An. coluzzii samples "M" in the "m_s" column of the associated metadata.
